# Correlation of Metabolic Syndrome with Redox Homeostasis Biomarkers: Evidence from High-Fat Diet Model in Wistar Rats

**DOI:** 10.3390/antiox12010089

**Published:** 2022-12-30

**Authors:** Danila Di Majo, Pierangelo Sardo, Giuseppe Giglia, Valentina Di Liberto, Francesco Paolo Zummo, Maria Grazia Zizzo, Gaetano Felice Caldara, Francesca Rappa, Giorgia Intili, Roelof Maarten van Dijk, Daniele Gallo, Giuseppe Ferraro, Giuditta Gambino

**Affiliations:** 1 Department of Biomedicine, Neuroscience and Advanced Diagnostics (BIND), University of Palermo, 90127 Palermo, Italy; 2 Postgraduate School of Nutrition and Food Science, University of Palermo, 90100 Palermo, Italy; 3 Euro Mediterranean Institute of Science and Technology, I.E.ME.S.T, 90139 Palermo, Italy; 4 Department of Biological, Chemical and Pharmaceutical Sciences and Technologies (STEBICEF), University of Palermo, Viale delle Scienze, 90128 Palermo, Italy; 5 ATeN (Advanced Technologies Network) Center, Viale delle Scienze, 90128 Palermo, Italy; 6 University of Palermo, 90100 Palermo, Italy; 7 Staburo GmbH, 81549 München, Germany

**Keywords:** oxidative stress, anti-oxidant barriers, glucose tolerance, lipid metabolism, non-alcoholic fatty liver disease, adipose tissue distribution

## Abstract

Metabolic Syndrome (MetS) is an extremely complex disease. A non-balanced diet such as high-fat diet (HFD) induces metabolic dysfunction that could modify redox homeostasis. We here aimed at exploring redox homeostasis in male Wistar rats, following 8 weeks of HFD, correlating the eventual modification of selected biomarkers that could be associated with the clinical manifestations of MetS. Therefore, we selected parameters relative to both the glucose tolerance and lipid altered metabolism, but also oxidative pattern. We assessed some biomarkers of oxidative stress i.e., thiols balance, lipid peroxidation and antioxidant barriers, via the use of specific biochemical assays, individuating eventual cross correlation with parameters relative to MetS through a Principal Component Analysis (PCA). The present study shows that 8 weeks of HFD induce MetS in rats, altering glucose and lipid homeostasis and increasing visceral adipose tissue, but also impairing the physiological antioxidant responses that could not counteract the oxidative stress condition. Crucially, cross-correlation analysis suggested that the assessment of specific oxidative stress parameters reported here can provide information comparable to the more widely acquired biomarkers of Mets such as glucose tolerance. Lastly, hepatic steatosis in association with the oxidative stress condition was also highlighted by histological analysis. This research will elucidate the fundamental impact of these oxidative stress parameters on MetS induced in the HFD rat model, tracing paths for developing prevention approaches.

## 1. Introduction

Metabolic Syndrome (MetS) is a clinical condition that affects more than a billion people worldwide and is of high importance to the healthcare system [1]. The main predisposing factors are: (i) hypercaloric and atherogenic diet, rich in saturated fatty acids and low in fibre; (ii) sedentary lifestyles and (iii) a genetic predisposition [1]. MetS is a multifactorial condition comprising overweight, visceral obesity, hepatic steatosis, dyslipidemia, hyperglycemia, insulin resistance, increased blood pressure, oxidative stress and inflammation, but could also be characterized by metabolic inflexibility that accounts for the inadequate capacity of the body to adapt to the different availability of nutrients leading to dangerous switches in substrate utilization [2,3,4,5,6,7,8].

Without proper clinical management, MetS can relapse when a patient developes type 2 diabetes, obesity and cardiovascular diseases, namely conditions that are associated with cognitive perturbations and other comorbidities [4,9,10,11]. Considering MetS pathogenesis as extremely complex and not yet fully elucidated, exploiting animal models of MetS will promote more in-depth knowledge of the phenomena and related prevention. Indeed, the experimental application of an obesogenic high-fat diet (HFD) has been reported to unbalance energy uptake and energy expenditure, leading to altered weight gain and metabolic profile from 8 weeks on [12,13,14]. Rats consuming an extreme high-fat diet showed increased plasma glucose levels and plasma lipid levels, also developing the glucose tolerance that impeded a proper utilisation of the glucose to establish homeostasis after a glucose challenge [15,16]. However, discrepancies have arisen among rodent models, dietary composition and chosen time course of extreme diets for assessment of MetS [17]. Studies compared and characterised the response to individual obesogenic diets in relation to dietary composition, since alternate diets could differently affect metabolic consequences differently [18]. Nonetheless, excess nutrient supply profoundly enhances oxidative stress and mitochondrial alteration, therefore inevitably driving—regardless of dietary composition itself—metabolic dysfunction during extreme diets. This is a condition that could be improved via supplementation with specific molecules such as short-chain fatty acids or N-acylethanolamines [19,20]. On this point, exploring the impact of oxidative stress and antioxidant barrier potential in MetS, induced in the HFD rat model, would suggest important insights and trace paths for developing prevention approaches [21]. The condition of oxidative stress is the consequence of an alteration in the plasma redox balance in which oxidant species prevail over antioxidant systems. There is a complex relationship between oxidative stress and metabolic syndrome that still deserves further investigation. It appears that oxidative stress may be both the cause and the consequence of obesity and other MetS-associated disorders [22]. Alterations in the metabolic state, such as those evident in metabolic syndrome, enhance the production of reactive oxygen species (ROS) that, if not completely neutralised by antioxidant systems, can lead to degeneration of functional structures, DNA, lipids and proteins, with accompanying alterations in cellular physiological processes. The overproduction of ROS can cause the oxidation of thiol groups with the formation of disulphide bridges, thus shifting the ratio in favour of the latter towards an arrangement with a more oxidised state. Oxidation to disulfide is a reversible process so it becomes important to monitor this ratio in order to increase antioxidant defences and shift the overall situation in favour of thiols [23]. The balance between thiols and disulfides is important for cellular processes such as programmed cell death, cell transduction mechanisms, enzymatic efficiency, detoxification processes and transcription processes [24]. The shift towards disulphides alters many physiological processes, making the body more susceptible to the development of metabolic [25], cardiovascular [26] and tumour diseases. Importantly, alterations of the described balance between oxidative stress parameters and antioxidant defences have been identified in rat models, both at the plasmatic level and in tissues, such as liver and brain. However, it still lacks a complete panel of redox homeostasis biomarkers associated with the clinical manifestations of MetS. Indeed, as shown in previous studies, the levels of plasmatic lipid peroxidation products are increased after 8 weeks [27] or 10 weeks [27,28] of high-fat diets in rats. At the same time, the liver of the HFD rat model exhibits hepatic lipid peroxidation and dysfunction [15]. High oxidative stress promotes the development of non-alcoholic fatty liver disease (NAFD), playing a key role in the progression of hepatic steatosis [29]. Such hepatic oxidative damage may also trigger extracellular matrix deposition and fibrosis in the liver, which is prevented by the supplementation of antioxidant-rich food. In this light, in the present study we aimed at investigating oxidative stress biomarkers and plasma antioxidant defences in MetS induced in male Wistar rats following 8 weeks of HFD. For this purpose, we employed four-week-old rats that were previously found to be more susceptible to oxidative stress and thus develop the MetS phenotype earlier with respect to older adults, guaranteeing a more time-saving and cost-effective rat model [30]. In detail, we first verified the induction of MetS after 8 weeks of HFD in rats by evaluating the rate of body weight gain, glucose tolerance, dyslipidemia and the relative amount of visceral adipose tissue. Then, via the use of specific biochemical assays, we assessed the prooxidant status via the analysis of hydroperoxides and lipoperoxides and the antioxidant defences. These functioned by evaluating thiolic groups and plasma antioxidant barriers, and to the best of our knowledge had never previously been applied. Interestingly, in this study we report comprehensive information obtained about the overall pattern of metabolic and oxidative alterations in MetS by means of a cross-correlation of body weight gain and biochemical markers, including glucose and lipid tolerance parameters, together with redox homeostasis parameters. Thanks to the PCA analysis, we were able to assess the influence of redox homeostasis variables on clinical manifestations of MetS. Lastly, we performed an ex vivo histological hepatic assay for the evaluation of NAFD. Our study, by employing a large sample size and focusing on novel redox homeostasis biomarkers in the HFD model, integrates this field of investigation into metabolic syndrome well. The present research will indeed shed more light on the condition of oxidative stress and on the importance of specific biomarkers in MetS, one of the most crucial factors when developing complications in diet-induced obesity in rats.

## 2. Materials and Methods

### 2.1. Animals and Diet Composition

Male Wistar rats (4 week-old, n = 40), weighing 240–260 g were provided by Envigo S.r.l (Indianapolis, Indiana). They were housed two per cage and maintained on a 12 h on/off cycle (8:00–20:00 h) at a constant temperature (22–24 °C) and humidity (50 ± 10%). During the acclimation period, animals were first fed with a standard chow diet providing providing 3.94 kcal/g and then divided into two homogenous groups with balanced weight. These groups were: fed with standard laboratory food (code PF1609, certificate EN 4RF25, Mucedola, Milan, Italy), or fed with HFD food with of 60% energy coming from fats (code PF4215-PELLET, Mucedola, Milan, Italy). The type of diet fed to animals to induce metabolic syndrome is a 60% fat-energy HFD. Fatty acids composition plays a crucial role in the regulation of metabolic phenotype. Saturated fatty acids (SFA) are more obesogenic and lead to larger significant metabolic alterations than unsaturated fatty acids. The lipid component of the HFD pellet comes for 9% from palm oil consisting of 50% saturated fat, 39% monounsaturated fat and 11% polyunsaturated fatty acids while the remaining fat comes from lard. The HFD is rich in saturated fatty acids in which the ratio of saturated/monounsaturated to polyunsaturated fatty acids is 4:3:1. The lipid quality consists of SFA/MUFA/PUFA in the ratios 1:1:3. The control group (NPD: normal pelletized diet) was fed with a low-fat diet represented by a standard rat chow (3.94 kcal/g, 3.18–55.50% carbohydrate, 22% protein, 3.50% lipid, 4.50% fiber). The lipid quality consists of SFA/MUFA/PUFA in the ratios 1:1:3. The second experimental group (HFD: high-fat diet) with a hypercaloric pelletized diet consisting of 34% fat, 23% protein, 38% carbohydrates and 5% fiber, until metabolic syndrome was induced, was assessed following criteria already established by the previous literature [14]. All rats had free access to food and water. Prior to starting the special diet, all animals were weighed. Additionally, blood samples from the caudal vein were obtained from a representative group of rats to collect the initial values of glucose, triglyceride (TG) and total cholesterol (TOT Chol) plasma concentrations, considered as the “T0” time. Animal care and handling throughout the experimental procedures were in accordance with the European Directive (2010/63/EU). The experimental protocols were approved by the animal welfare committee of the University of Palermo and authorised by the Ministry of Health (Rome, Italy; Authorization Number 14/2022-PR). Pellet composition for both HFD and NPD are reported in Table 1.

### 2.2. Body Weight Gain and Food Intake

Since the HFD treatment started, animals were weighed once a week over 8 weeks for the evaluation of body weight gain until the metabolic syndrome was induced. Furthermore, the food intake was evaluated in the two experimental groups. To do so, the chow was weighed before and after being delivered to the cage twice a week. The remaining chow was weighed, and food intake was determined by the difference between the weight of the chow that was given to the animals and the weight of the remaining chow.

### 2.3. Determination and Quantification of Metabolic Syndrome

In order to identify the onset of metabolic syndrome, one time point was selected after the beginning of the special diets based on literature [14,15], i.e., 8 weeks when peripheral insulin resistance has developed. At this point, body weight gain due to HFD was analysed together with biochemical parameters from representative plasma samples such as TG, TOT Chol, HDL, GTT. Finally, we assessed by micro-computed tomography (micro-CT) scans adipose tissue volumes in the visceral and subcutaneous depots by expressing the results as volumes relative to body weight. Once differences were individuated between HFD and NPD groups, analyses were conducted on rats in the two groups at “T1” time, confirmed as the point of induction of metabolic syndrome.

#### 2.3.1. Glucose Tolerance Test

Glucose Tolerance Test (GTT), a diagnostic tool for diabetes and indicator of metabolic efficiency and insulin resistance, was conducted at T1 (8 weeks of HFD vs. NPD). The GTT was performed taking into account the guidelines reported by Bowe et al. [31]. After an overnight fast, a drop of blood was collected from the tail vein and expressed on a blood glucose test strip (Glucotest, Pic) to obtain a baseline value using a Glucometer (Glucotest, Pic). Then, a solution of 20% glucose was i.p. injected (2 g/kg body weight), and blood glucose levels were measured at 15, 30, 60, 90 and 120 min after glucose injection. The area under the curve (AUC) was calculated for HFD and NPD groups, considering the blood glucose levels (mg/dL) per unit of time as in [32]. The PG-AUC was calculated by trapezoidal approximation of PG levels. PG levels at x min were defined as PG (x), and PG-AUC was calculated as follows:

(1) $$\mathrm{PG}\;\mathrm{AUC}\;(\mathrm{mg}\cdot h/\mathrm{dL})=\frac{\mathrm{PG}\;\left(o\right)+\;\mathrm{PG}\;\left(30\right)\;\times \;2+\;\mathrm{PG}\left(60\right)\times \;3+\;\mathrm{PG}\left(120\right)\times \;2}{4}$$

#### 2.3.2. Plasma Samples

Before (T0) and after the induction of MetS by high-fat-diet (HFD) (T1), the blood samples were collected by the caudal vein of the tail. For each animal, an amount of about 1 mL of blood was collected in microcuvettes with lithium heparin (MicrovetteCB 300 mL, Lithium Heparin, orange UE code, Fisher Scientific, Waltham, MA, USA), centrifuged at 3000 rpm for 10 min, and the resulting plasma was analyzed or stored at −80 °C until analysis.

#### 2.3.3. Biochemical Analyses

In the plasma samples, glucose, triglycerides (TG), total cholesterol (TC), low-density lipoprotein cholesterol (LDL-C), high-density lipoprotein cholesterol (HDL-C) concentrations were quantified by commercial kits using the Free Carpe Diem device (FREE^®^ Carpe Diem; Diacron International, via Zircone 8, 58100, Grosseto (GR), Italy). Glucose levels are detected since glucose, in the presence of glucose oxidase (GOD), is oxidized to gluconic acid and hydrogen peroxide. By the action of peroxidase (POD), hydrogen peroxide reacts to form a colored compound that can be detected by a spectrophotometer at 510 nm. Triglycerides levels are detected because triglycerides, in the presence of lipoprotein lipase (LPL), are hydrolyzed to fatty acids and glycerol. Glycerol, by the action of glycerol kinase (GK) and glycerol-3-phosphatases (GPO), reacts to form hydrogen peroxide. By the activity of peroxidase (POD), hydrogen peroxide reacts to form a colored compound detected by a spectrophotometer at 546 nm. Total cholesterol levels are detected since esterified cholesterol, in the presence of cholesterol esterase (CHE), is hydrolyzed in cholesterol and fatty acids. By the action of cholesterol oxidase (CHOD), cholesterol is oxidized to form hydrogen peroxide. Hydrogen peroxide, by the activity of peroxidase (POD), reacts to form a colored compound that can be detected by a spectrophotometer at 510 nm. HDL or LDL cholesterol levels are detected based on surfactant activity which, by eliminating VLDL, HDL or LDL cholesterol and chylomicrons, allows the remaining HDL or LDL cholesterol to react to form a colored compound detected by a spectrophotometer at 600 nm. The data are always expressed in mg/dL.

#### 2.3.4. Oxidative Stress Parameters

Plasma redox balance was assessed using Diacron kits whose scientific validity has been amply demonstrated and is reported in the literature [33,34,35,36]. To assess the pro-oxidant status, the D-ROM (Reactive Oxygen Metabolites) and the LP-CHOLOX test were carried out, the former assessing the levels of hydroperoxyl free radicals and the latter the levels of circulating lipid peroxides and in particular oxidised cholesterol. LP-CHOLOX test is useful for assessing atherosclerotic risk and preventing the onset of cardiovascular disease. In the plasma samples, hydroperoxides, lipoperoxides and oxidized cholesterol were measured by commercial kits using the Free Carpe Diem device (FREE^®^ Carpe Diem; Diacron International, Italy). In the d-ROMs Test, ROMs (primarily hydroperoxides) levels are detected based on the ability of transition metals to catalyze, in the presence of peroxides, the formation of free radicals that are then trapped by an alkylamine. The alkylamine reacts to form a colored radical that can be detected by a spectrophotometer at 505 nm. Data are expressed in arbitrary units, namely Carratelli units (UCARR). The normal range of the test results was 250–300 U.CARR (Carratelli Units), where 1 UCARR corresponds to 0.08 mg/dL of H_2_O_2_ [37]. In the LP CHOLOX Test, LP CHOLOX (lipoperoxides and oxidized cholesterol) levels are detected based on peroxides ability to facilitate the oxidation of Fe^2+^ to Fe^3+^ which binds to an indicator mixture forming a colored complex detected by a spectrophotometer at 505 nm [38,39]. The results are expressed in mEq/L.

#### 2.3.5. Plasma Antioxidant Status

As for the plasma antioxidant status, the BAP-TesT (Biological Antioxidant Potential), it measures substances of an exogenous nature (ascorbate, tocopherols, carotenoids and bioflavonoids) and substances of an endogenous nature (bilirubin, uric acid and proteins) in plasma that have antioxidant potential and are capable of counteracting radical species. It is an indicator of the body’s ability to defend itself against ROS. The OXY Adsorbent Test and the SH Test were used for the analysis. In the samples plasmatic thiols groups and plasma barrier were measured by commercial kits using the Free Carpe Diem device (FREE^®^ Carpe Diem; Diacron International, via Zircone 8, 58100, Grosseto (GR), Italy). In the -SHp Test, thiols groups (-SH) levels are detected based on the ability of thiols groups to develop in a buffered solution, reacting with a chromogenic mixture, a coloured complex photometrically detectable at 410 nm [40]. According to the literature there is a correlation between OXY adsorbent test and FRAP but the mechanism by which they exert their antioxidant power is different [41,42]. The OXY adsorbent test measures non-enzymatic antioxidants and their ability to adsorb radical species [41]. In the OXY-Adsorbent Test, the antioxidant power of the plasma barrier (OXY-Adsorbent) is detected by measuring the ability of the barrier to oppose the massive oxidant action of hypochlorous acid (HClO). The entity of the plasma barrier—which includes mucopolysaccharides, amino acids, proteins and all the antioxidants not active from the chemical point of view—are measured by difference between sample absorbance and a blank made by HClO [43]. The results are expressed as mmol HClO/mL of sample. In the BAP Test, the biological antioxidant potential (BAP) of plasma barrier is detected based on the ability of all antioxidants active from the chemical point of view to reduce a coloured solution of ferric ions (Fe^3+^) to ferrous ions (Fe^2+^), generating a bleaching photometrically detectable at 505 nm. Data are expressed as mmol/L [44].

### 2.4. Micro-Computed Tomography Scans

Micro-computed tomography (micro-CT) scans were performed to assess the volumes of the visceral adipose tissue (VAT) and subcutaneous adipose tissue (SAT). A representative sample of rats from each group were randomly selected at T1 and anesthetized with 5% isoflurane. Transverse micro-CT images of the abdomen from L1 to L5 were obtained by the micro-CT scanner Quantum FX µCT (Perkin-Elmer, Hopkinton, MA, USA) in the animal facility of ATeN Center—University of Palermo. Voltage was set at 50 kV and current was set at 200 μA and the images were captured over a 4.5 min interval. Analysis of micro-CT images was conducted with AnalyzePro software (AnalyzeDirect, Overland Park, KS, USA). Visceral and subcutaneous adipose tissue was segmented in the sagittal plane and measurements of volume obtained with the Region of Interest mode. Tissue volumes are expressed relative to body mass. Procedures were performed following standardised protocols [45].

### 2.5. Histological Analyses

At the end of the experimental procedures, animals were euthanized using 2% isoflurane anaesthesia followed by cervical dislocation. Hepatic samples were collected for histological analyses. Samples were immediately stored in paraformaldehyde for 48 h, then were moved to a 20% PBS/sucrose solution and after 1 week to 10% PBS/sucrose solution. At last, the solution was removed and samples were stored at −80 °C. Liver tissue sections (5 µm) were obtained from cryostat and stained with haematoxiln and eosin. Following staining, the slides were observed with an optical microscope (Microscope Axioscope 5/7 KMAT, Carl Zeiss, Oberkochen, Germany) connected to a digital camera (Microscopy Camera Axiocam 208 color, Carl Zeiss). For the steatosis evaluation, a semiquantitative analysis was performed by two independent observers in a high-power field (HPF) (magnification 400×) and repeated for 10 HPFs.

### 2.6. Statistical Analyses

Statistical analysis was performed by GraphPad Prism 9.02 (San Diego, CA, USA). Body weight gains, food intake and blood glucose levels (GTT) were analysed via a two-way repeated measures (RM) ANOVA, followed by Bonferroni post-hoc test for significant differences for intra-subject comparisons, considering the effect of “time”, “diet” and their interaction in the experimental groups. Values of TG, TOT Chol, AUC, oxidative stress biomarkers, micro-CT and histological evaluations were compared by an unpaired Student’s *t*-test for differences between means and represented by scattered bar graphs. Differences were considered significant when *p* < 0.05. The results are presented as the mean ± standard error of the mean (S.E.M.), apart from GTT values at 0 and 120 min presented as box and whiskers plots. Correlation matrix was calculated using R version 4.2.2 and visualized using the R package “corrplot”. Missing values for correlation matrix were handled with ‘pairwise complete observation’. Principal component analysis (PCA) was calculated and visualized using the R package “made4.” For each variable in the PCA the mean of the remaining animals (regardless of group) was added instead of missing values.

## 3. Results

### 3.1. Effects of HFD Treatment on Body Weight and Food Intake

**Figure 1:**
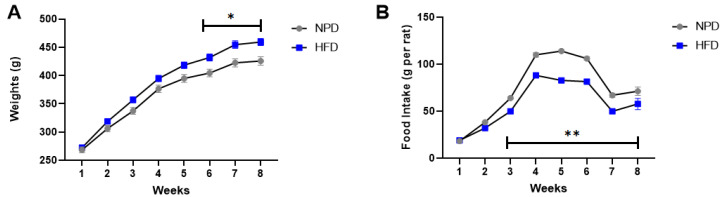
Body weights and food intake in NPD and HFD experimental groups for 8 weeks (T1) of HFD diet. (**A**) Weights. Body weight gains (g) difference between NPD and HFD groups in 8 weeks of HFD. (**B**) Food intake. Food intake (g per cage) difference in NPD and HFD groups in 8 weeks of HFD. Statistical significance indicated for (*) *p* < 0.05 and (**) *p* < 0.001.

At T0 animals were balanced in basal conditions and their average weights were comparable when subdivided into experimental groups (NPD: 256.85 ± 18.96 g, HFD: 260.38 ± 17.29 g). In the first week of exposure to the new diet, the food intake was not different between HFD and NPD (NPD: 36.54 ± 18.27 g, HFD: 38.33 ± 19.16 g). At T1, after 8 weeks of HFD, body weight gains were compared via a two-way repeated measures ANOVA. Significant differences in body weight were found in HFD vs. NPD for time (F_(7,238)_ = 1185, *p* < 0.001), diet (F_(1,34)_ = 7.37, *p* = 0.013) and their interaction (F_(7,238)_ = 7.63, *p* < 0.001). Post-hoc test revealed marked increases in HFD vs. NPD from 6th to 8th week, as indicated in Figure 1A, with a mean % weight increase >70% at 8th week (HFD: 75.44 ± 7.29 g). Furthermore, food intake was reduced in HFD vs. NPD as evidenced by a two-way RM ANOVA for time (F_(7,119)_ = 353.8, *p* < 0.001), diet (F_(1,17)_ = 112.6, *p* < 0.0001) and their interaction (F_(7,119)_ = 10.36, *p* < 0.0001); and Bonferroni post hoc test, as in Figure 1B.

### 3.2. Effects of HFD Treatment on Glucose Tolerance

**Figure 2:**
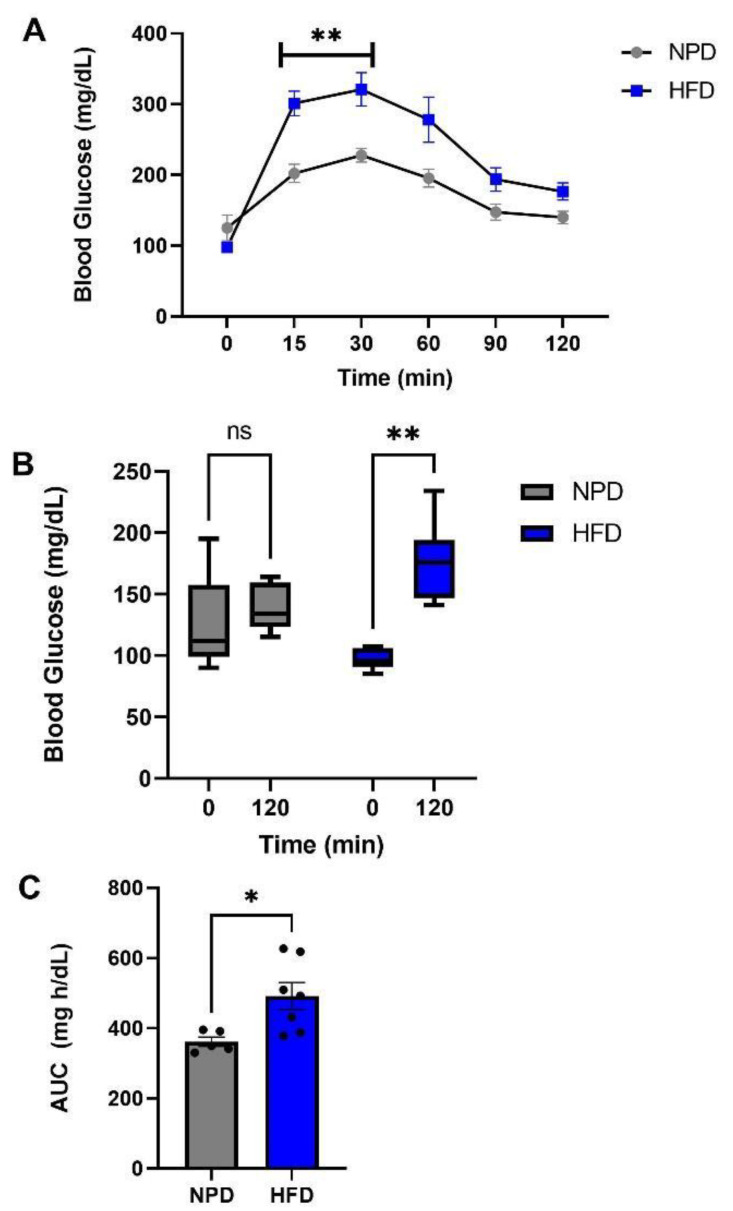
GTT test in NPD and HFD experimental groups after 8 weeks (T1) of HFD. (**A**) Glucose levels in GTT. Plasma glucose levels (mg/dL) differences between NPD and HFD groups for different times of GTT. (**B**) Glucose levels at 120 min. Plasma glucose levels (mg/dL) differences between NPD and HFD groups until 120 min after injection. (**C**) Area under the curve (AUC). Plasma glucose levels (mg/dL) per unit of time (h) difference between NPD and HFD groups. Statistical significance indicated for (*) *p* < 0.05 and (**) *p* < 0.01.

GTT test performed 8 weeks after the beginning of the special diet outlined significant differences between HFD and NPD. Indeed, a two-way RM ANOVA, followed by a Bonferroni post-hoc test performed on GTT at 0, 15, 30, 60, 90 and 120 min revealed marked differences in plasma glucose levels for time (F_(5,50)_ = 44.25, *p* < 0.001), diet (F_(1,50)_ = 8.44, *p* = 0.0157) and their interaction (F_(5,50)_ = 6.46, *p* = 0.001) in HFD compared to NPD groups (Figure 2A). In agreement with other data in the literature [46], no significant differences are observed between the two groups in fasting glucose levels while there are different trends over time after i.p. glucose administration. In detail, glucose administration modulated plasma glucose levels in HFD rats reaching a significant increase from 15 min versus NPD. A reduction was then observed in the NPD group at 60 min that returned to basal values at 120 min after injection. Whereas, HFD rats increased plasma glucose levels until 120 min, not returning to baseline, as shown in Figure 2B, for time (F_(1,10)_ = 14.08, *p* = 0.0038), diet (F_(1,10)_ = 0.22, *p* = 0.064) and their interaction (F_(1,10)_ = 6.51, *p* = 0.028). Lastly, we evaluated the AUC, as described in methods section, by the Student’s *t*-test that highlighted an increase in HFD vs. NPD group (t = 2.75, df = 10, *p* = 0.025, Figure 2C).

### 3.3. Effects of HFD Treatment on Lipid Profile and on the Distribution of Adipose Tissue

At T0 plasma samples were collected from a representative group of rats before starting the diet to describe the basal metabolic profile. Results are presented in Table 2.

**Figure 3:**
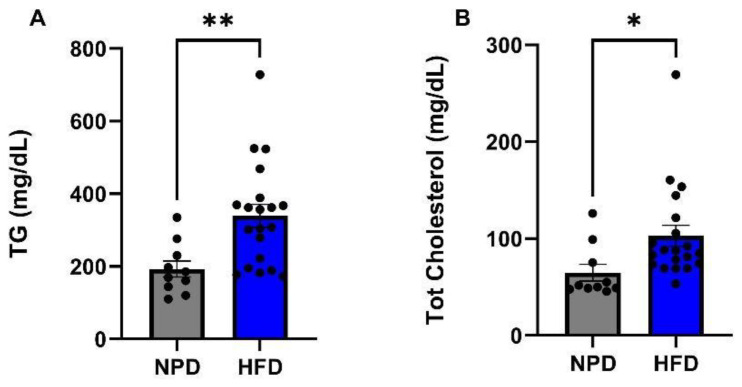
Lipid profile in NPD and HFD experimental groups after 8 weeks (T1) of HFD. (**A**) Triglycerides (TG). Plasma TG levels (mg/dL) differences between NPD and HFD groups after 8 weeks of HFD. (**B**) Total cholesterol. Plasma total cholesterol levels (mg/dL) differences between NPD and HFD groups after 8 weeks of HFD. Statistical significance indicated for (*) *p* < 0.05 and (**) *p* < 0.01.

After 8 weeks of HFD, plasma samples were collected at T1 from HFD and NPD groups to assess eventual changes in lipid profile in the condition of metabolic syndrome. The Student’s *t*-test performed on the plasma levels of TG pointed out a significant increase in HFD vs. NPD group (t = 3.05, df = 28, *p* = 0.0049, Figure 3A), with a mean % increase of TG in HFD of 75.44 ± 7.29%. Furthermore, total cholesterol was markedly augmented in HFD vs. NPD (t = 2.26, df = 28, *p* = 0.031, Figure 3B). In agreement, the plasma levels of HDL were remarkably decreased in HFD group (mean: 33.74 ± 1.529 mg/dL) with respect to NPD (mean: 22.65 ± 1.374 mg/dL), as shown by an unpaired *t*-test (t = 4.54, df = 28, *p* < 0.0001).

**Figure 4:**
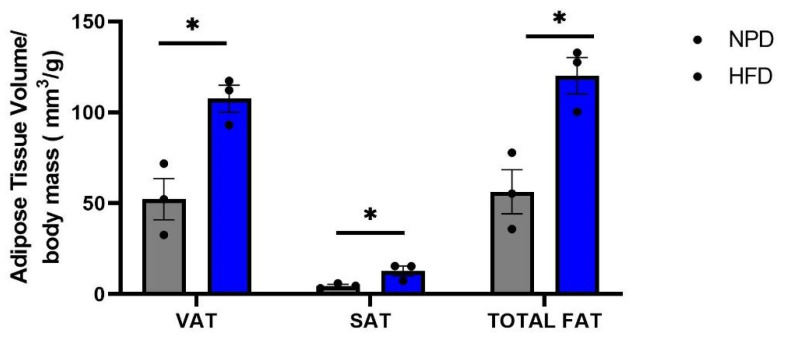
Transverse micro-CT quantification. Visceral adipose tissue (VAT), subcutaneous adipose tissue (SAT) and total adipose abdominal tissue (Total FAT) expressed relative to body mass in NPD and HFD rats. Data are the means ± S.E.M. Statistical significance indicated for (*) *p* < 0.05.

Lastly, micro-CT analysis revealed that the total fat depots were significantly increased in HFD rats compared to NPD animals, as evidenced by an unpaired *t*-test (t = 4.053, df = 4, *p* = 0.0154; Figure 4). Interestingly, HFD rats showed also a significant increase in VAT and SAT volume/body mass ratio in comparison with NPD animals (respectively, t = 4.087, df = 4, *p* = 0.0150 and t = 4.053, df = 4, *p* = 0.0454; Figure 4).

### 3.4. Effects of HFD on Plasma Redox Homeostasis Biomarkers

**Figure 5:**
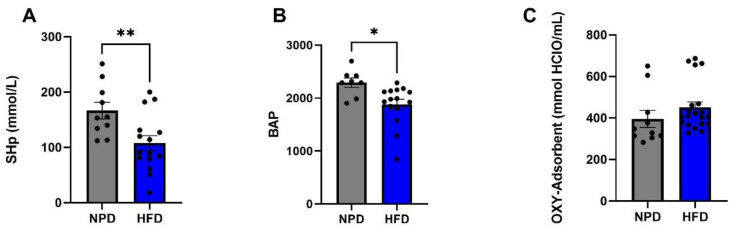
Antioxidant status in NPD and HDF experimental groups after 8 weeks (T1) of HFD. (**A**) SHp Test. Thiols groups levels (mmol/L) difference between NPD and HFD groups after 8 weeks of HFD. (**B**) BAP Test. Biological antioxidant potential levels (mmol/L) difference between NPD and HFD groups after 8 weeks of HFD. (**C**) OXY-Adsorbent Test. The antioxidant power of the plasma barrier levels (mmol HClO/mL) difference between NPD and HFD groups after 8 weeks of HFD. Statistical significance indicated for (*) *p* < 0.05 and (**) *p* < 0.01.

**Figure 6:**
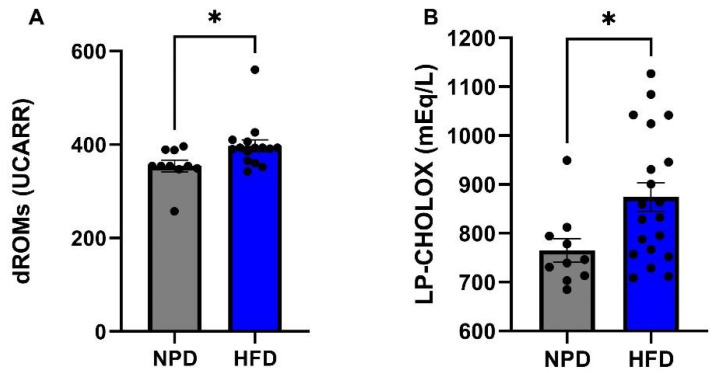
Pro-oxidant status in NPD and HFD experimental groups after 8 weeks (T1) of HFD. (**A**) dROMs Test. Differences in ROMs (primarily hydroperoxides) levels (UCARR) between NPD and HFD groups after 8 weeks of HFD. (**B**) LP-CHOLOX Test. Differences in LP-CHOLOX (lipoperoxides and oxidized cholesterol) levels (mEq/L) between NPD and HFD groups after 8 weeks of HFD. Statistical significance for (*) *p* < 0.05.

The antioxidant and pro-oxidant status was evaluated at time T1, 8 weeks after the start of the diet, on plasma samples from the HFD group of rats and the NPD group to explore the plasma redox balance in metabolic syndrome. Statistical analyses revealed that HFD markedly reduced antioxidant capacity of animals with respect to NPD. In particular, an unpaired *t*-test was conducted on mean values of SHp and BAP tests showing a reduction in HFD vs. NPD (respectively: t = 2.88, df = 28, *p* = 0.0084 and t = 2.73, df = 28, *p* = 0.0125 as in Figure 5A,B). No significant differences emerged analysing the OXY Adsorbent levels (Figure 5C). Lastly, statistical significance emerged from the analysis conducted on mean values of the pro-oxidant status, i.e., dROMs and LP-Cholox levels, revealing an increase in HFD compared to NPD group (respectively: t = 2.28, df = 28, *p* = 0.0321 and t = 2.41, df = 28, *p* = 0.022, as in Figure 6A,B).

### 3.5. Spearman’s Correlation Matrix of MetS and Oxidative Stress Parameters

**Figure 7:**
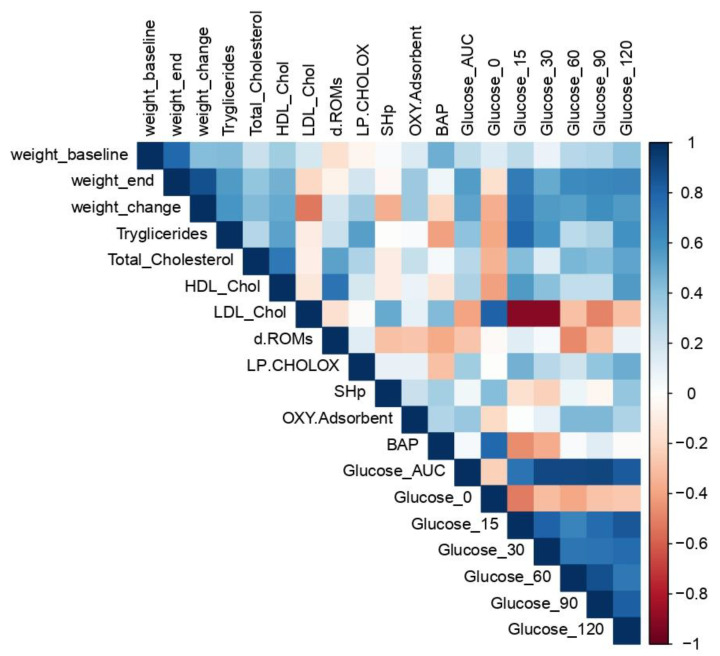
Correlation matrix of measured variables. Heat maps representing Spearman correlation matrices between biochemical and body weight variables. Missing values were handled with ‘pairwise complete observation’.

A Spearman correlation matrix was performed to evaluate the relations between the clinical variables considered to characterise the MetS and the parameters used to identify the oxidative stress condition. Correlations emerged between different parameters assessed in MetS as indicated in Figure 7. In particular, Spearman’s correlation analysis shows a strong positive correlation between the altered lipid profile present in the condition of induced MetS and the pro-oxidant status revealed by D-ROM test and LP-CHOLOX test. More precisely, a medium-intensity correlation can be observed between total cholesterol and the D-ROM test (ρ = 0.53) and between total triglyceride levels and the LP-CHOLOX test (ρ = 0.54), while a negative correlation can be observed with the pro-oxidant status assessed by the BAP test (ρ = −0.41). The change in body weight, on the other hand, shows a negative correlation with the analysis of thiol groups (ρ = −0.36) and a positive correlation with the pro-oxidant status assessed by LP CHOLOX test and OXYadsorbent Test (respectively ρ = 0.35 and ρ = 0.36). With regard to the alteration of glucose tolerance assessed by means of the blood glucose curve, the analysis shows a medium positive correlation (ρ = 0.49) between glucose levels assessed at 120 min and the LP-CHOLOX test.

### 3.6. Principal Component Analysis

**Figure 8:**
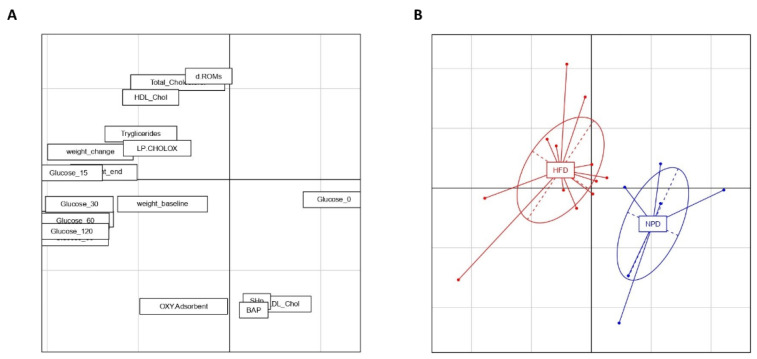
Principal component analysis (PCA) of measured variables in the HFD and NPD groups. PCA with PC1 (35.58%) on the x-axis and PC2 (16.75%) on the y-axis. (**A**) The loading of the 2 principal components, where the further the parameters are from the midpoint the larger their impact is on the 2 principal components. (**B**) The individual animals for each group along the two axes. Distribution of the two groups is significantly different along PC1, with individual comparisons showing significant differences.

PCA was carried out on the centered and scaled data of biochemical and redox homeostasis variables in MetS (Figure 8). The first two principal components (PCs) together account for 52.33% of the variance in the data (PC1 = 35.58% and PC2 = 16.75%). A significant separation between the two experimental groups can be seen along PC1 thanks to a one-way ANOVA and post-hoc test (F_(1,16)_ = 17.93, *p* = 0.0006). Furthermore, non-significant differences emerged between groups along PC2 by a one-way ANOVA followed by a post-hoc test (F_(1,16)_ = 3.52, *p* = 0.078). The separation between groups along PC1 can be attributed not only to the variation in MetS of parameters related to glucose tolerance, to body weight gain and lipid homeostasis, but also to the redox homeostasis biomarkers analyzed in the present study. In particular, we can observe in Figure 8A that redox homeostasis parameters like dROMS test are strictly correlated with lipid profile biomarkers forming a cluster that in turn negatively correlates with the cluster formed by SHp and BAP.

### 3.7. Effects of HFD on Hepatic Steatosis

**Figure 9:**
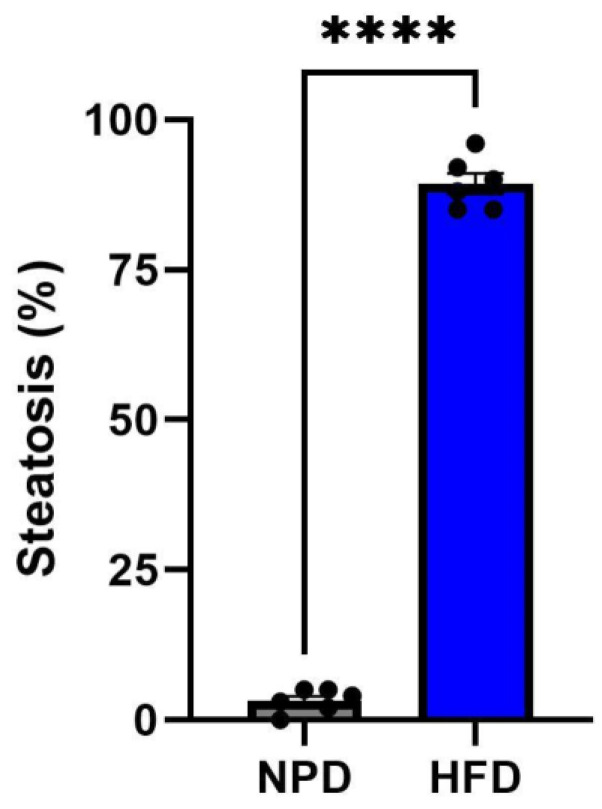
Histological evaluation of hepatic steatosis. Differences in hepatic steatosis (%) between NPD and HFD groups. **** for *p* < 0.0001.

**Figure 10:**
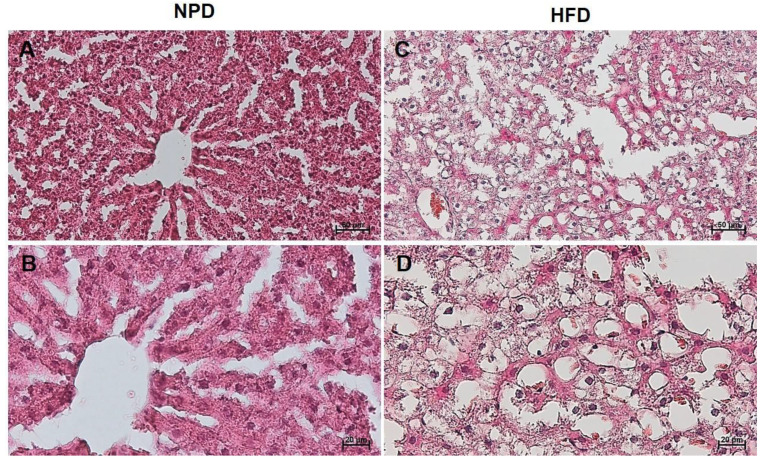
Histological features of liver tissue of NPD and HFD groups. Representative images of hematoxylin and eosin staining of liver tissue (**A**,**B**: NPD; **C**,**D**: HFD). (**A**,**C**): magnification 200×, scale bar 50 µm. (**B**,**D**): magnification 400×, scale bar 20 µm.

The steatosis evaluation performed on liver samples of the NPD group showed an almost absent steatosis (average percentage of 3.2 ± 0.8) compared to the cases of the HFD group in which steatosis was found to be high (average percentage of 89.3 ± 1.7), as shown by a significant unpaired *t*-test (t = 44.97, df = 10, *p* < 0.0001, Figure 9 and Figure 10).

## 4. Discussion

The dangerous liaison between oxidative stress and MetS has been recently hinted at. An in-depth understanding of this relationship could provide bases for monitoring the influence of oxidative stress on MetS key symptoms, namely central obesity, hypertension, glucose tolerance and dyslipidemia, but also could have face validity in the development of effective preventive approaches [47]. With this in mind, the present research considers other parameters than traditional (MDA) to assess oxidative stress in MetS exploiting a well-structured HFD rat model that has been widely considered ideal for this field of studies [14]. The initial evaluation of metabolic parameters before starting the diet (T0) are in accordance with what has been previously reported for male Wistar rats of the same age and weight [15,46]. Our large series of data obtained, with respect to other investigations [46] have revealed that HFD induced the complete clinical manifestations of MetS in terms of increased body weight and reduced food intake, but also inducing glucose tolerance and dyslipidemia. Even though no standardized criteria have been established to diagnose the development of MetS in animal models, according to Rodriguez et al. [14] it is necessary that at least three parameters are increased by a well-defined percentage with respect to normally-fed rats. In our study, the criteria considered to detect the induction of MetS were: the increased percentages of TG levels, of VAT and SAT volume/body mass ratio and body weight gain, together with glucose tolerance. Indeed, after 8 weeks of special diet, HFD rats clearly showed a severely higher body weight than normal rats, concurrently decreasing their food intake. In other words, even if they ate less then NPD rats, they were obese. This can be explained by considering the effect of leptin, a hormone produced by the adipose tissue regulating feeding behavior through action at the hypothalamic level where it reduces the perception of food reward and increases the release of satiety signals that result in reduced food intake [48]. Also, leptin is implicated in the modified oxidative and inflammatory pattern that could account for numerous effects induced by MetS [49,50,51]. At the same time, it also exerts its action at the peripheral level through receptors on pancreatic beta cells, resulting in inhibition of insulin secretion and increased sense of satiety [48,52,53].

Furthermore, our data did not show any difference in basal fasting blood glucose levels between HFD and NPD, as revealed also by some authors [46]. However, GTT outlined that, notwithstanding the normal basal levels recorded, HFD determined a marked hyperglycemia 15 min after glucose administration, with respect to NPD. These higher blood glucose levels in HFD were not compensated at 120 min of GTT, in contrast to glucose levels of NPD rats that were restored to baseline. This condition correlates positively with the production of lipoperoxide radicals, assessed by LP-Cholox. This let us state that after 8 weeks of HFD glucose tolerance has been induced in terms of normal basal glucose levels and after 120 min >140 mg/dL, similarly to criteria applied in clinical practice [32]. Moreover, dyslipidemia was found after 8 weeks of HFD since TG, total cholesterol and LDL values were remarkably increased compared to NPD, whereas HDL were dampened. The data obtained are in line with the literature [14,46]. This suggests that the increase in plasma cholesterol and triglycerides in rats on the HFD compared to control rats is indicative of hepatic lipogenesis characterised by the development of NAFD, a condition that fuels the formation of acetyl CoA with consequences for the cellular redox state. NAFD could explain the hypercholesterolemia that we highlighted, that could be a consequence of hepatic insulin resistance leading to reduced synthesis of ApoB and thus VLDL [29]. In addition, our findings from imaging CT revealed a different distribution of adipose tissue in HFD rats with respect to controls. Particularly, they show a marked increase in visceral and subcutaneous fat, adding evidence to further confirm the induction of MetS according to the reported criteria [14]. The hepatic steatosis detected in the liver of HFD rats impairs the ability of adipocytes to accumulate lipids, leading to ectopic lipid redistribution, as already observed by other authors [45]. Consequently, this condition leads to systemic metabolic dysfunction, that could be based on metabolic inflexibility, among the others, a condition that was found to be counteracted by several molecules [54,55].

This experimental picture corroborates the efficacy of HFD protocol here applied for further evaluation of how oxidative parameters are modified. In HFD rats, after 8 weeks, evaluation of plasma redox balance showed an inadequate antioxidant physiological response to counteract the oxidative stress condition induced by the developed MetS. Our assessment of antioxidant status revealed that values of thiolic groups and antioxidant barriers, consisting of endogenous and exogenous plasma antioxidants, are strongly impaired in MetS, since we found that HFD reduces both SHp and BAP parameters versus controls, and these parameters negatively correlate with the alterations in plasma triglycerides induced by metabolic syndrome.

In particular, thiols belong to the mercaptan family and are functional groups of many plasma and cellular components such as albumin, antioxidant enzymes, transcriptional factors that regulate many important physiological processes [23]. The major thiol/disulfide redox couple in human plasma is cysteine (Cys) and its disulfide form, cystine (CySS). Since extracellular thiol/disulfide redox environments are highly regulated in healthy individuals, our data of reduced levels of thiolic groups in HFD rats are in line with a shifted balance towards disulfide compounds. This shifted balance towards disulfide has been correlated with chronic metabolic, autoimmune and degenerative diseases [56]. Oxidation to disulfide is a reversible process so it becomes important to monitor this ratio in order to increase antioxidant defences and shift in favour of thiols [23]. Furthermore, the results from the BAP test indicate decreased levels in HFD rats versus NPD. This could be due to the physiological compensation triggered in MetS in order to counteract the excessive free radicals, ultimately inducing a depletion of own antioxidant defences. As for the OXY-adsorbent test, no significant differences emerged after HFD treatment. This suggests a lack of the plasma reducing potential.The oxidative biomarkers i.e., dROMS and LP-Cholox were markedly enhanced in HFD with respect to normally-fed rats, in agreement with a lower antioxidant status and with previous findings showing increased plasmatic lipid peroxidation products [27]. Intriguingly, we observed that in the correlation matrix these biomarkers positively correlate with glucose and lipid variables in MetS. The observed high levels of hydroperoxides demonstrated by dROMS test, influences the signalling transduction pathways via the alteration of kinases and phosphatases proteins and favours the oxidation of thiolic groups to disulfide bridges, supporting our data from SH p levels [57]. Hydroperoxydes together with lipoperoxidation also were found to determine modifications that could evolve in hepatic oxidative damage in humans and animals [57]. This hepatic oxidative damage could increase levels of nitric oxide (NO) and advanced protein oxidation products (APOP) and augment plasmatic activities of common hepatic dysfunction markers, such as the aspartate transaminase (AST), alanine aminotransferase (ALT) and alkaline phosphatase (ALP) enzymes [15]. The pro-oxidant status here revealed in HFD rats was not compensated by endogenous antioxidant barriers that were decreased, suggesting an impairment of redox homeostasis. Hypotesizeably, according to previous literature, the detrimental effects of MetS on redox balance might be associated with the decreased superoxide dismutase (SOD), catalase (CAT), glutathione peroxidase (GPx) and glutathione (GSH) observed [58,59,60]. Excess ROS were indeed found to reduce antioxidant molecules such as GSH and SOD but also to plays a key role in the conversion of NAFD into hepatic cirrhosis [60,61,62]. Even if the main goal of our study was to evidence the possible correlation between less-investigated parameters related to antioxidant barriers and pro-oxidant status with MetS, further investigations studies on homeostatic redox enzymes would corroborate the importance of the endogenous antioxidant defences on the development of MetS.

The importance of the redox homeostasis parameters assessed in the present research is corroborated by a cross-correlation analysis that validates the use of these assays in MetS condition since they appear to be correlated with the most common metabolic variables altered. Also, the PCA analysis revealed that pro-oxidant status biomarkers show an integrated behaviour that negatively correlates with antioxidant barrier parameters in MetS.

Considering this, our findings strongly support the close relationship between MetS and oxidative stress in line with previous literature adding knowledge that consumption of HFD is correlated with specific oxidative stress biomarkers in many animal experimental models and in patients with clinical conditions related to obesity [46,63]. Noteworthy, impaired metabolic pathways in MetS could become the driving force towards oxidative stress [64]. This could also represent a silent threat to cognitive performance, together with several other intracellular players that could be implicated in the regulation of neuronal excitability and neurodegeneration [65,66,67,68]. With this in mind, our research provides solid bases for the individuation of coherent and uniform biomarkers that correlate redox homeostasis with metabolic and tissue modifications induced by MetS, exploiting a large sample size of male Wistar rats. Our experimental choice indeed fills the gap of standardising MetS rat models, still not present enough in previous studies [69], as a key way to assess oxidative stress alterations in this pathophysiological condition, and thus strengthening the translational validity of this preclinical model.

## 5. Conclusions

In conclusion, this study shows that, in a male Wistar rat model, MetS condition was induced following 8 weeks of HFD with a hepatic profile typical of NAFD, which is associated with an altered plasma redox balance shifted towards high levels of hydroperoxides. The oxidative biomarkers here applied could be of great interest to assess redox homeostasis since they are correlated with the clinical condition, assessing the effects of antioxidant-based therapies on MetS and eventual NAFD degeneration.

## Figures and Tables

**Table 1:** Pellet compositions are reported for both HFD and NPD. (SFA) Saturated Fat Acid, (MUFA) Monounsaturated Fatty Acid, (PUFA) Polyunsaturated Fatty Acid.

	Pellet HFD (PF4215)	NPD (PF1609)
Energy (Kcal/Kg)	5500–6000	3947
Fat Total (g/100 g)	60	3.50
SFA (g/60 g)	30	0.7
MUFA (g/60 g)	23	0.8
PUFA (g/60 g)	7	2
Crude Protein (g/100 g)	23	22
Carbohydrates (Starch g/100 g)	38	35.18
Sugar (g/100 g)	-	5.66
Fibre (g/100 g)	5	4.5
Ash (g/100 g)	5.50	7.5
Vitamin A (IU)	8400	19.533
Vitamin D3 (IU)	2100	1260

**Table 2:** Basal metabolic parameters in plasma samples at T0.

Basal Biochemical Parameters (T0)	Mean ± S.D.
Glucose (mg/dL)	180.51 ± 38.71
Triglycerides	166.94 ± 41.83
Tot Cholesterol	66.67 ± 7.86
HDL Cholesterol	36.93 ± 8.2

## Data Availability

The data presented in this study are available on reasonable request from the corresponding author.
